# Sex Effects in Mouse Prion Disease Incubation Time

**DOI:** 10.1371/journal.pone.0028741

**Published:** 2011-12-13

**Authors:** Shaheen Akhtar, Adam Wenborn, Sebastian Brandner, John Collinge, Sarah E. Lloyd

**Affiliations:** Medical Research Council Prion Unit and Department of Neurodegenerative Disease, University College London Institute of Neurology, London, United Kingdom; Ohio State University, United States of America

## Abstract

Prion disease incubation time in mice is determined by many factors including PrP expression level, *Prnp* alleles, genetic background, prion strain and route of inoculation. Sex differences have been described in age of onset for vCJD and in disease duration for both vCJD and sporadic CJD and have also been shown in experimental models. The sex effects reported for mouse incubation times are often contradictory and detail only one strain of mice or prions, resulting in broad generalisations and a confusing picture. To clarify the effect of sex on prion disease incubation time in mice we have compared male and female transmission data from twelve different inbred lines of mice inoculated with at least two prion strains, representing both mouse-adapted scrapie and BSE. Our data show that sex can have a highly significant difference on incubation time. However, this is limited to particular mouse and prion strain combinations. No sex differences were seen in endogenous PrP^C^ levels nor in the neuropathological markers of prion disease: PrP^Sc^ distribution, spongiosis, neuronal loss and gliosis. These data suggest that when comparing incubation times between experimental groups, such as testing the effects of modifier genes or therapeutics, single sex groups should be used.

## Introduction

Prion diseases or transmissible spongiform encephalopathies involve the conversion of the normal host PrP^C^ to an abnormal form, PrP^Sc^. They are a group of fatal neurodegenerative diseases that affect both humans and animals and include sheep scrapie, bovine spongiform encephalopathy (BSE) in cattle and Creutzfeldt-Jakob disease (CJD) in humans [1]. Human prion diseases have three distinct aetiologies: sporadic, inherited and acquired. Sporadic CJD is the most common form of human prion disease accounting for ∼85% of all cases and to date there is no known cause. 15% of cases are inherited or familial and are caused by mutations in the prion gene (*PRNP*). Prion diseases are also infectious and are therefore transmissible (acquired). Human prion diseases have been transmitted through medical procedures (iatrogenic) such as the administration of contaminated pituitary hormones and the use of infected neurosurgical electrodes. Following human exposure to BSE contaminated material, a new acquired prion disease, variant CJD (vCJD), was recognised.

Irrespective of aetiology and host, prion diseases are distinguished by long, clinically silent incubation periods and characteristic neuropathology such as PrP^Sc^ accumulation, spongiform change (vacuolation), gliosis and neuronal death. Prion disease incubation time in experimental models such as inbred mice is highly reproducible under standard conditions. However, there is also considerable variation that is determined by several factors including host genetic background, prion strain and route of inoculation. The main genetic determinant is variation in *Prnp* itself that results in two major isoforms of PrP: *Prnp^a^* (108L, 189T) and *Prnp^b^* (108F, 189V) that are associated with short and long incubation times respectively [2]–[5]. Comparison of incubation times from *Prnp^a^* inbred lines shows a difference of over 100 days between the shortest and longest incubation time strains suggesting the involvement of additional genetic loci [6]. Several quantitative trait loci mapping studies have identified regions of the genome that contain genetic factors that influence incubation time in mice [7]–[10] and further fine mapping has identified individual candidate genes [11]–[13].

Prion strains are associated with conformational and glycosylation differences in PrP^Sc^ and result in different incubation times even in the same host [14]. Indeed, the characteristic incubation period, neuropathology and biochemistry form the basis on which different prion strains are defined [15], [16].

The most common route for the inoculation mice with prions is either intracerebral or intraperitoneal. Delivery of prions directly to the central nervous system rather than to the periphery results in shorter incubation times [17], [18].

Sex has also been implicated in influencing prion disease incubation time in mice. This effect has been suggested by a few studies, however, these have not been comprehensive and have largely focused on only one strain of mice or prions [19]–[22]. Variation in the choice of model and prion strain have also compounded the difficulty of replicating these findings resulting in equivocal data and a confusing picture. Sex effects have also been described in human prion diseases with females exhibiting a two year earlier age of onset than males for vCJD after stratification by birth cohort [19]. Survival times for females in both CJD and vCJD have also shown to be longer increasing from four to five months and twelve to fourteen months respectively [23].

In order to address the effect of sex on prion disease incubation time in mice we have compared transmission data for males and females gathered at the MRC Prion Unit over the last ten years. This includes data from twelve different inbred lines of mice inoculated with at least two prion strains representing both mouse adapted scrapie (Chandler/RML) and mouse adapted BSE. We show that sex can have a profound influence on incubation time, however, this is not a general effect and is not seen in all mouse strains but rather is highly specific for given combinations of mouse model and prion strain.

## Results

### Prion transmissions to inbred lines

Groups of males and females from twelve different inbred lines of mice (including two sub-strains of C57BL/6) were inoculated intra-cerebrally with various strains of prions. All mouse lines were challenged with a strain of mouse-adapted scrapie and mouse-adapted BSE. These were generally Chandler/RML scrapie prions (I856) and C57BL/6 passaged BSE (I874), however, ME7 scrapie prions (I9459) and mouse-passaged BSE (MRC2, I9468) were also transmitted in some cases (Table 1). The inbred lines of mice represent a range of genetic backgrounds and incubation times ranging from a “short” incubation time strain such as NZW/OlaHsd to a “long” incubation time strain such as MAI/Pas. Most strains are *Prnp^a^* allele mice, however, JU/FaCt is a *Prnp^b^* strain and MAI/pas is a *Prnp^c^* strain [6]. Most mouse lines are standard laboratory inbred lines with the exception of PERA/Kamei, CAST/Ei, PWK/Pas and MAI/Pas that are derived from wild-trapped animals [24].

**Table 1:** Prion incubation times.

	RML/Scrapie (I856)			ME7 (I9459)			Mouse Passaged BSE (I874)			MRC2 (I9468)		
	Male	Female	p-val	Male	Female	p-val	Male	Female	p-val	Male	Female	p-val
C57BL/6JOlaHsd	146±1 (20)	140±1 (21)	0.005				173±1 (22)	180±1 (20)	0.0002			
C57BL/6J				168±1 (10)	164±0 (13)	0.001				173±1 (5)	177±0 (10)	0.0001
FVB/NHsd	136±2 (15)	127±1 (18)	0.0007	154±1 (14)	149±2 (9)	0.037	155±2 (13)	157±2 (19)	ns	147±3 (8)	152±2 (14)	0.013
NZW/OlaHsd	110±1 (16)	107±2 (22)	0.006				133±1 (15)	132±1 (13)	ns			
RIIIS/J	136±1 (17)	134±1 (17)	ns				164±2 (18)	169±2 (21)	ns			
SJL/OlaHsd	121±1 (19)	122±1 (18)	ns				152±3 (15)	158±2 (22)	ns			
SWR/OlaHsd	135±1 (19)	135±2 (17)	ns				153±2 (13)	158±1 (14)	ns			
SM/J	129±1 (27)	137±1 (20)	<0.0001				171±1 (17)	173±1 (12)	ns			
CAST/Ei	193±5 (5)	186±3 (3)	ns				184±9 (3)	180±6 (6)	ns			
PERA/Kamei	148±1 (13)	153±2 (13)	ns				191±6 (5)	206±10 (12)	ns			
PWK/Pas	224±5 (8)	214±10 (4)	ns				181±3 (5)	185±4 (8)	ns			
JU/FaCt	317±4 (9)	310±4 (15)	ns				354±13 (6)	398±83 (2)	ns			
MAI/Pas	372±4 (10)	348±4 (9)	ns				329±9 (4)	321±7 (7)	ns			

Incubation times are given for prion transmissions following intra-cerebral inoculation and are displayed as the mean ± sem given in days with the number of animals per group given in parentheses. RML and Me7 represent two distinct strains of mouse-passaged scrapie and mouse BSE and MRC2 represent two different preparations of mouse passaged BSE. For all prion strains, all groups of mice were inoculated with 1% brain homogenate made from the same stock of 10% brain homogenate. All RML/scrapie and mouse passaged BSE transmissions were carried out in a conventional animal facility and ME7 and MRC2 transmissions were carried out in a specific-pathogen-free facility. Where data were normally distributed groups were analysed statistically using a t-test. Where one or both of the groups did not pass a normality test, a non-parametric Mann-Whitney test was used. ns denotes not significant (P>0.05).

Incubation times for all transmissions are shown in table 1. For 8 of the 12 mouse lines no significant differences were seen between males and females with either Chandler/RML or mouse-passaged BSE prions. For SM/J and NZW/OlaHsd significant differences were seen between males and females (p<0.0001 and p = 0.006 respectively) with Chandler/RML, but not mouse-passaged BSE, prions. For SM/J mice the mean incubation time was 6% longer in females as compared to males, however, the situation was reversed for NZW/OlaHsd where the mean incubation time in females was 3% shorter than in males.

**Figure 1:**
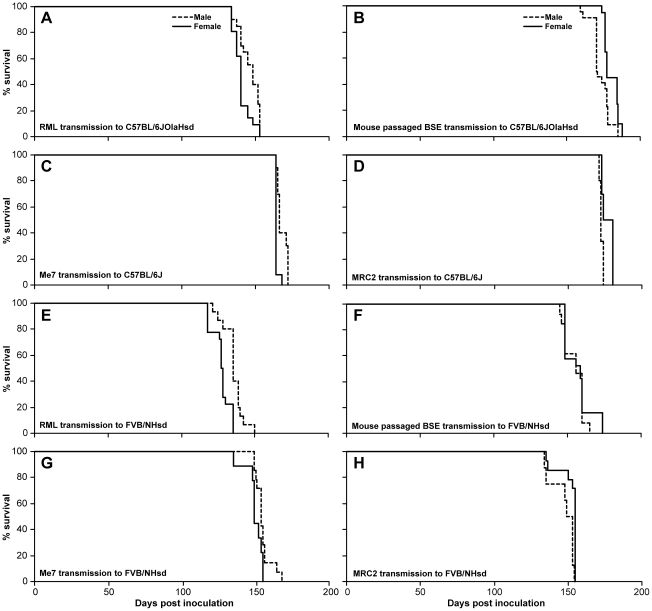
C57BL/6 and FVB/N survival curves. Survival curves for prion transmissions to C57BL/6J (A–D) and FVB/NHsd (E–H) mice. A and E, Chandler/RML prions; B and F, mouse passaged BSE prions; C and G, ME7 prions; D and H, MRC2 prions. % survival is shown on the y-axis and days post inoculation is shown on the x-axis. All data are significant (P<0.05) except for mouse-passaged BSE transmission to FVB/NHsd. Detailed values are given in Table 1.

Two lines of mice, C57BL/6 and FVB/NHsd, showed significant sex differences with multiple prion strains (Table 1, Figure 1). For C57BL/6 (C57BL/6JOlaHsd or C57BL/6J) highly significant sex differences were observed for all four prion strains. For both mouse-adapted scrapie prion strains the mean incubation time in females was 4 or 2% (Chandler/RML and ME7 respectively) shorter than in males (p = 0.05 and p = 0.001) while for both mouse-passaged BSE prion strains this was reversed with the female mean incubation time being longer than for males (4 and 2%, p = 0.0002 and p = 0.0001 for I874 and MRC2-I9468 respectively). FVB/NHsd displayed a significant sex effect with Chandler/RML (p = 0.0007), ME7 (p = 0.037) and MRC2-I9478 (P = 0.013) but not for mouse passaged BSE (I874). These effects are in the same direction as for C57BL/6 with the mean incubation time in females being shorter for mouse-adapted scrapie strains (7 and 3% for Chandler/RML and ME7 respectively) and the mean incubation times in females being 3% longer with MRC2-I9468.

### PrP^C^ levels

**Figure 2:**
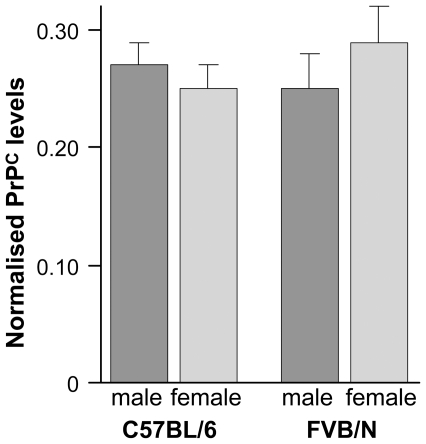
PrP^C^ levels for male and female C57BL/6 and FVB/N mice. PrP^C^ levels (ug/ml) were determined in triplicate using 10% (weight/volume) brain homogenate for male (n = 3) and female (n = 3) mice of each strain in a PrP specific ELISA. Data are shown normalised by total protein content (ug/ml and ×1000) as determined by a BCA assay (mean ± standard deviation). No significant differences were seen between males and females or between strains.

In experimental mouse models, the level of PrP^C^ expression is inversely correlated to incubation time. The shortest incubation times are seen in transgenic models over-expressing mouse PrP [25]. While *Prnp* null mice are resistant to prion disease, one copy of *Prnp* restores susceptibility but with an increased incubation time relative to wild type controls [26]. To determine whether differences in endogenous PrP^C^ levels could explain the sex effects seen in C57BL/6 and FVB/N mice, we measured total PrP^C^ in 10% brain homogenates for both males and females using a PrP specific ELISA [27]. No differences were seen between the sexes or between mouse strains (Figure 2).

### Neuropathology

**Figure 3:**
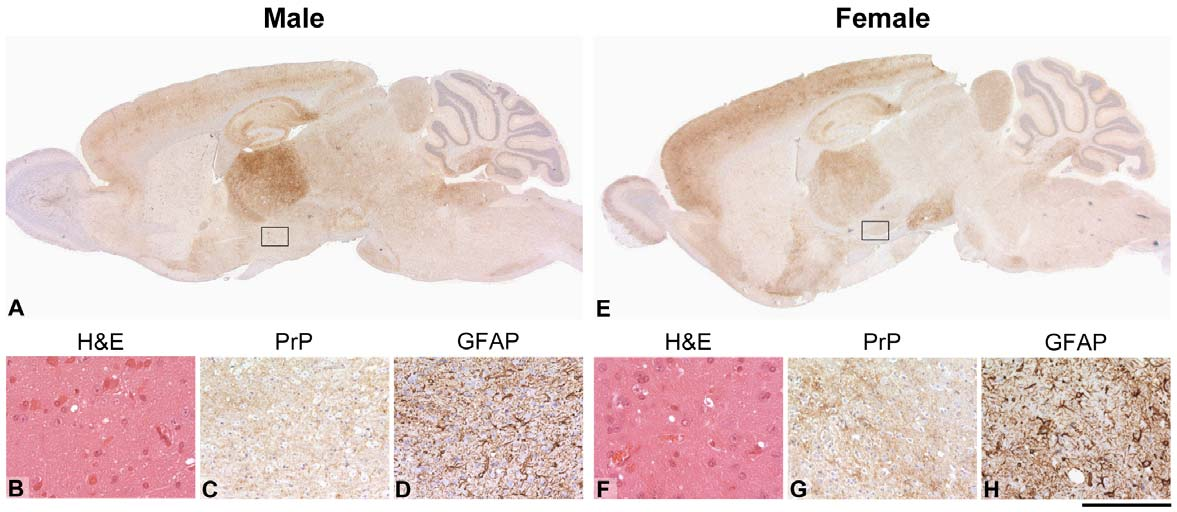
Histological features of Chandler/RML inoculated male and female FVB/N mice. Comparison of histological features between male FVB/N (left panel, A–D) and female FVB/N mice (right panel, E–H) inoculated with Chandler/RML prions. Panels A and E show the PrP^Sc^ distribution in a cross section of the brain and panels B–D and F–H are higher power views of the hypothalamus (boxed area). (B and F) mild spongiosis (C and G) synaptic deposition of PrP^Sc^ (D and H) gliosis. Overall, the pattern of PrP^sc^ distribution, spongiosis or gliosis, shows no difference between both groups. Scale bar corresponds to 2 mm (A, E), 80 µm (B, F) or 160 µm in all other panels.

**Figure 4:**
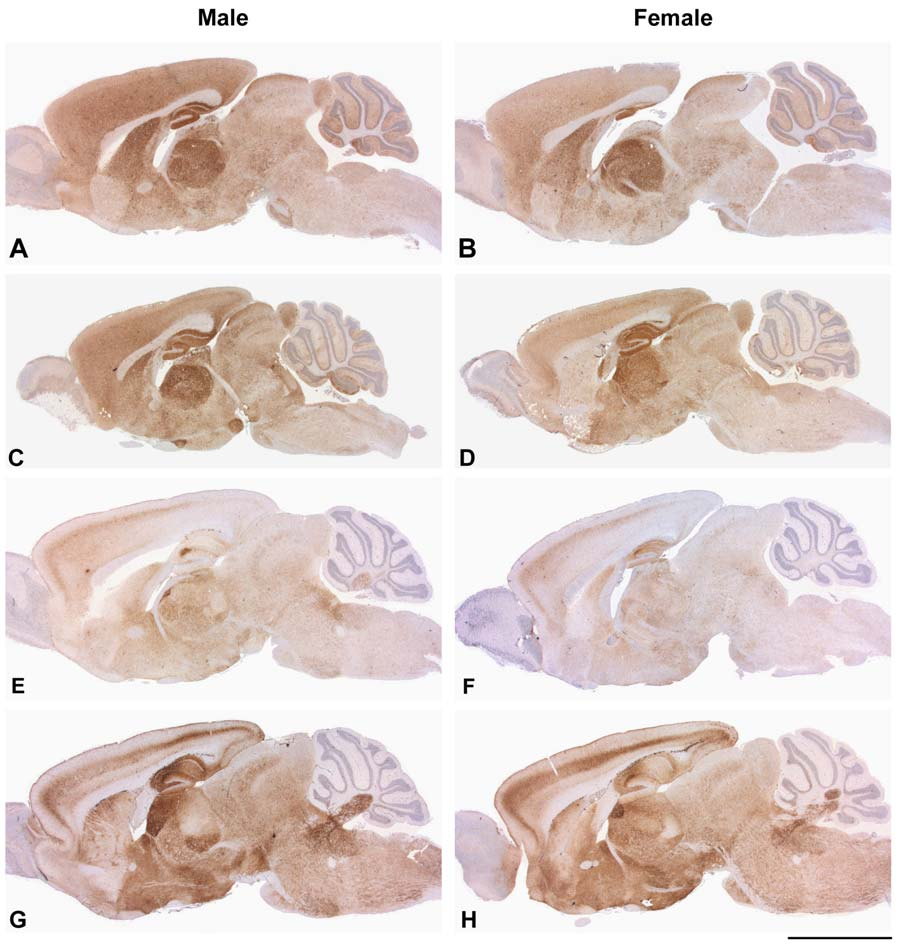
PrP^Sc^ distribution in the brains of C57BL/6 and FVB/N mice. Comparison of prion protein staining between male (A, C, E, G) and female (B, D, F, H) mice inoculated with ME7 prions (A–D) and MRC2 prions (E–H). A, B and E, F represent C57BL/6 mice and C, D and G, H represent FVB/N mice. Overall, the pattern of PrP^Sc^ distribution shows no difference between male and females and is characteristic of the prion strain used. Scale bar corresponds to 2.5 mm for all panels except for C and D where it corresponds to 3 mm.

To establish whether regions of the brain showed differential susceptibility between males and females we compared the pattern of PrP^Sc^ deposition, spongiosis, gliosis and neuronal loss in groups of males and females (5–14 per group) for C57BL/6 and FVB/NHsd for Chandler/RML, ME7 and MRC2 prion strains. Endocrine differences have been implicated as determinants of sex effects in prion disease [19]. The pituitary was unavailable for analysis as this is generally lost during the removal of the brains, however, the hypothalamus was looked at in detail. For all groups no significant differences were seen between males and females or between the mouse strains (Figure 3 and figures 4 and S1). For both males and females the patterns of neuropathology were characteristic of the prion strains used.

### Prion strain typing

**Figure 5:**
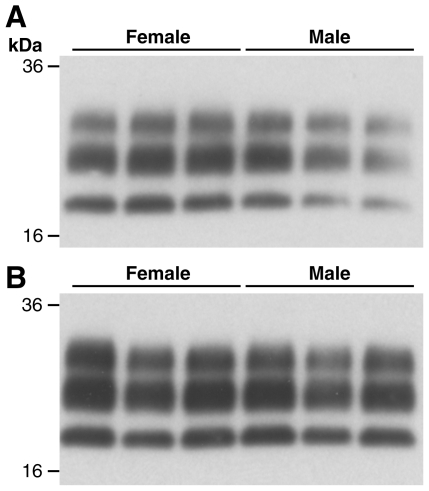
Western blot of PrP^Sc^ from the brains of C57BL/6 and FVB/N mice following Chandler/RML transmission. Western blot of proteinase-K treated 10% w/v brain homogenates (n = 3 for both males and females) immunoblotted with anti-PrP monoclonal antibody ICSM-35 (D-Gen Ltd, UK). (A) C57BL/6 mice (B) FVB mice. The PrP^Sc^ from both male and female brains is characteristic of the RML scrapie prion strain and no sex differences are seen.

In addition to neuropathological differences, the other important feature for strain discrimination are the molecular characteristics of the protease-resistant prion protein (PrP^Sc^) as shown by immunoblotting. We compared the pattern of PrP^Sc^ in end stage brains from males and females (3 per group) for C57BL/6 and FVB/NHsd for Chandler/RML, ME7 and MRC2 transmissions. In all cases the characteristic PrP^Sc^ pattern for the prion strain was preserved and no differences were observed between males and females (Figure 5, S2 and S3).

## Materials and Methods

### Mice

Commercially available inbred mouse lines were obtained from Harlan, UK (Bicester, UK) or the Jackson Laboratory (Bar Harbor, Maine, USA). MAI/Pas and PWK/Pas mice were a gift from Jean-Louis Guénet (Institute Pasteur, Paris); JU/FaCt were a gift from Bruce Cattanach (MRC Harwell, UK) and CAST/Ei were obtained from the Mary Lyon Centre (MRC Harwell, UK).

### Prion strains

All inocula were prepared as 1% (weight/volume) prion infected brain homogenate in PBS made from the brains of terminally sick mice. The mouse adapted scrapie prion strain Chandler/RML (gift of A. Aguzzi, Institute of Neuropathology, University of Zurich, Zurich) was generated by a single passage in CD-1 Swiss mice [7]. A source of the ME7 mouse adapted prion strain was obtained from the Institute of Animal Health, UK and further passaged once in C57BL/6JOlaHsd mice. BSE tissues were originally obtained from the Veterinary Laboratories Agency, UK and passaged in inbred mice as previously described [28]. In brief, both BSE preparations were derived from a pool of five brainstems from naturally occurring cases of BSE. This material was passaged twice in C57BL6/JOlaHsd mice to produce the inoculum I874. This was further passaged in SJL/OlaHsd mice to produce the strain MRC2 (I9468) [28].

### Prion inoculation and phenotyping

Mice were anaesthetized with isofluorane/O_2_ and inoculated intra-cerebrally into the right parietal lobe with 30 µl of a 1% prion infected brain homogenate as previously described [7]. Mice were examined daily for neurological signs of prion disease and were culled once a definitive diagnosis had been made or earlier if showing signs of distress or loss of up to 20% of body weight. Diagnostic criteria for clinical prion disease were as previously described [29]. Non-specific early signs include agitation, aggression, unusual gait and slight weight loss and more specific early indicators include erect ears, rigid tail, un-groomed appearance, slight hunched posture and clasping of hind limbs when lifted. Definitive diagnosis was made on observation of one confirmatory sign of prion disease and animals were culled at this point. Confirmatory signs included ataxia, generalised tremor, loss of righting reflex, limb paralysis, extensive pilo-erection or sustained hunched posture. Incubation time was calculated retrospectively and defined as the number of days from inoculation to the onset of clinical signs. All sex difference comparisons were from experiments that were performed at the same time with the same inoculum with the same technicians recording neurological observations. All procedures were conducted in accordance with institutional and national regulations and standards on animal welfare. Experiments were approved by the MRC Prion Unit Animal Ethical Review Committee and carried out under UK Home Office licences PPL 80/1639 and PPL 70/6454.

### Immunohistochemistry

Mouse brains were fixed in 10% buffered formal saline (BFS) and prion infectivity was inactivated by incubation in 98% formic acid for one hour. Tissues were paraffin wax embedded, sectioned and stained as previously described [29]. In brief, sections were stained with haematoxylin and eosin (H&E) for general examination and determination of spongiosis and neuronal loss. Prion deposition was visualised with anti-PrP monoclonal antibody ICSM35 (D-Gen Ltd, UK) and gliosis was determined with an anti-glial fibrillary acid protein (GFAP) antibody (Dako UK, Ltd).

### Western blotting

10% w/v brain homogenates in D-PBS were prepared by ribolysing, benzonase treated, proteinase K digested (50 µg/ml of proteinase K for 1 h at 37°C) and western blotted as described previously [30]. Blots were probed with the anti-PrP monoclonal antibody ICSM-35 (D-Gen Ltd, UK) in conjunction with an alkaline-phosphatase-conjugated anti-mouse IgG secondary antibody (Sigma) and developed in chemiluminescent substrate (CDP-Star; Tropix Inc).

### Quantification of PrP^c^ by enzyme linked immunosorbent assay (ELISA)

Endogenous levels of PrP^C^ were determined in 10% brain homogenates by ELISA as previously described [27]. PrP^C^ was captured by anti-PrP monoclonal antibody ICSM18 (D-Gen Ltd, UK) and detected using biotinylated anti-PrP monoclonal antibody ICSM35 (D-Gen Ltd, UK) and streptavidin-horseradish peroxidise conjugate (Dako UK, Ltd). Total protein concentration was determined with a Pierce BCA Protein Assay kit (Thermo Scientific) according to the manufacturer's instructions.

### Statistical analysis

Statistical tests were carried out using GraphPad InStat (GraphPad Software, Inc, California, USA). Where the group incubation times passed a normality test (Kolmogorov-Smirnov), data were evaluated using a two-tailed t-test. Where the normality test was not passed the non-parametric Mann-Whitney test was used.

## Discussion

Sex effects have been implicated in prion disease incubation time for many years, however, to date, these data have been equivocal with few conclusions drawn. We have now presented a comprehensive study with 12 different inbred lines of mice all of which were challenged with a mouse-adapted scrapie strain of prions (Chandler/RML) and mouse-adapted BSE (I874). In addition, two mouse lines (C57BL/6 and FVB/N) were challenged with ME7 and the mouse-adapted BSE strain, MRC2. Our data show that sex can have a significant effect on incubation in some instances. However, this is highly specific to the mouse and prion strain combination. 8 of the 12 mouse strains used showed no sex differences with either prion strain and two mouse strains (NZW and SM) only showed a sex effect with the Chandler/RML prion strain. C57BL/6 and FVB/N were the only mouse strains to show sex differences with both mouse adapted scrapie and BSE. For these two mouse lines, females had a shorter incubation time than males when challenged with either Chandler/RML or ME7 but this was reversed following inoculation with BSE derived prions. This is broadly in agreement with previous studies showing that: female C57BL/6N have a shorter incubation time following inoculation with ME7 [19]; female *Prnp^b^* mice (VM/Dk, IM/Dk and MB/Dk) have a shorter incubation time than males following challenge with the mouse adapted scrapie strain; shorter incubation time for female NZW mice with Chandler/RML but not ME7 prions [21], [22]. The opposite finding was reported by Abiola *et al* in C57BL/6J mice inoculated with ME7 [20]. Analysis of combined data from 117 titration experiments with ten mouse strains and seven different murine scrapie strains concluded that female mice had on average a nine day shorter incubation time [31]. Based on our data, we suggest that it is not appropriate to pool data from different mouse and prion strains, however, it is possible that the data of McLean and Bostock broadly agree with our findings due to the predominance of C57 mice and the ME7 prion strain in their data set [31].

Few studies have looked at the effect of sex on prion disease incubation time following inoculation with BSE. On primary passage of BSE to C57 and RIII mice no sex differences were observed although an X chromosome-cytoplasm interaction was observed in the F1 backcross generation [10]. This contrasts with the findings of Abiola *et al* who described a highly significant increase in C57BL/6J and DBA/2J females on inoculation with BSE [20]. Although both these studies used C57BL mice they were a different sub-strain with C57BL/FaBtDk and C57BL/6J used by Manolakou *et al* and Abiola *et al* respectively. The extent of the similarity between these two sub-strains has not been characterised but it is possible that this may be sufficient to explain the contradictory findings. In our present study we looked at mouse-passaged BSE and where significant differences were observed, females showed an increase in incubation times relatives to males. It may not be appropriate to compare data from BSE primary passages with data from murine BSE strains as the presence of a species barrier and subsequent strain adaptation may significantly alter the sex effects.

Sex differences have also been reported for age of onset and disease duration of the human prion diseases, vCJD and sporadic CJD [19], [23]. Kingsbury *et al* also reported an increased incubation time in female B.10.AKM, C3H/SwSn and C3H/D1Sn mice following inoculation with mouse-passaged sporadic CJD strain K.Fu [21].

In this study we have used the number of days from inoculation to the onset of clinical signs as our measure of incubation time. It is also possible to measure incubation period as the time from inoculation to the time of death and to measure the duration of the disease. Using these alternative data it is possible that other sex differences may be observed.

The reason for the observed sex differences is still unresolved. In this study we have shown that there is no difference in the endogenous levels of PrP^C^ between males and females that show differences in incubation time with a range of prion strains. We have also shown that there are no detectable differences between males and females in terms of PrP^Sc^ deposition in the brain and the pattern and extent of spongiosis, gliosis and neuronal loss. No differences were seen in prion strain types between males and females as determined by PrP^Sc^ pattern by immunoblotting. Loeuillet *et al* investigated the effect of androgens on incubation time by using castrated mice [19]. Castration negated the sex effect after intracerebral inoculation but not following intraperitoneal inoculation suggesting that while androgens may play a role other factors such as differential inactivation of genes on the X chromosome are also likely to be important.

For some mouse inbred lines and prion strain combinations there is a highly significant difference in incubation time with a mean difference of up to 7%. While this effect is not seen with all mouse lines, it is of particular importance in C57BL/6 and FVB/N strains as these are commonly used for the generation of knockout and transgenic mouse models. We therefore recommend that when testing the effect of a modifying gene or therapeutic agent on prion disease incubation time it is essential to use single sex groups.

## Supporting Information

Figure S1 **Histological features of Chandler/RML inoculated male and female C57BL/6 mice.** Comparison of histological features between male C57BL/6/(left panel, A–D) and female C57BL/6 mice (right panel, E–H) inoculated with Chandler/RML prions. Panels A and E show the PrP^Sc^ distribution in a cross section of the brain and panels B–D and F-H are higher power views of the hypothalamus (boxed area). (B and F) mild spongiosis (C and G) synaptic deposition of PrP^Sc^ (D and H) gliosis. Overall, the pattern of PrP^Sc^ distribution, spongiosis or gliosis, shows no difference between both groups. Scale bar corresponds to 2 mm (A, E), 80 µm (B, F) or 160 µm in all other panels.
(JPG)

Figure S2 **Western blot of PrP^Sc^ from the brains of C57BL/6 and FVB/N mice following Me7 transmission.** Western blot of proteinase-K treated 10% w/v brain homogenates (n = 3 for both males and females) immunoblotted with anti-PrP monoclonal antibody ICSM-35 (D-Gen Ltd, UK). (A) C57BL/6 mice (B) FVB mice. The PrP^Sc^ from both male and female brains is characteristic of the Me7 scrapie prion strain and no sex differences are seen.
(TIF)

Figure S3 **Western blot of PrP^Sc^ from the brains of C57BL/6 and FVB/N mice following MRC2 transmission.** Western blot of proteinase-K treated 10% w/v brain homogenates (n = 3 for both males and females) immunoblotted with anti-PrP monoclonal antibody ICSM-35 (D-Gen Ltd, UK). (A) C57BL/6 mice (B) FVB mice. The PrP^Sc^ from both male and female brains is characteristic of the MRC2 prion strain and no sex differences are seen.
(TIF)
