# Supplementary figures and images for: Sex Effects in Mouse Prion Disease Incubation Time
Source: PLoS One. 2011 Dec 13;6(12):e28741. doi: 10.1371/journal.pone.0028741 (PMC3236759; doi:10.1371/journal.pone.0028741)

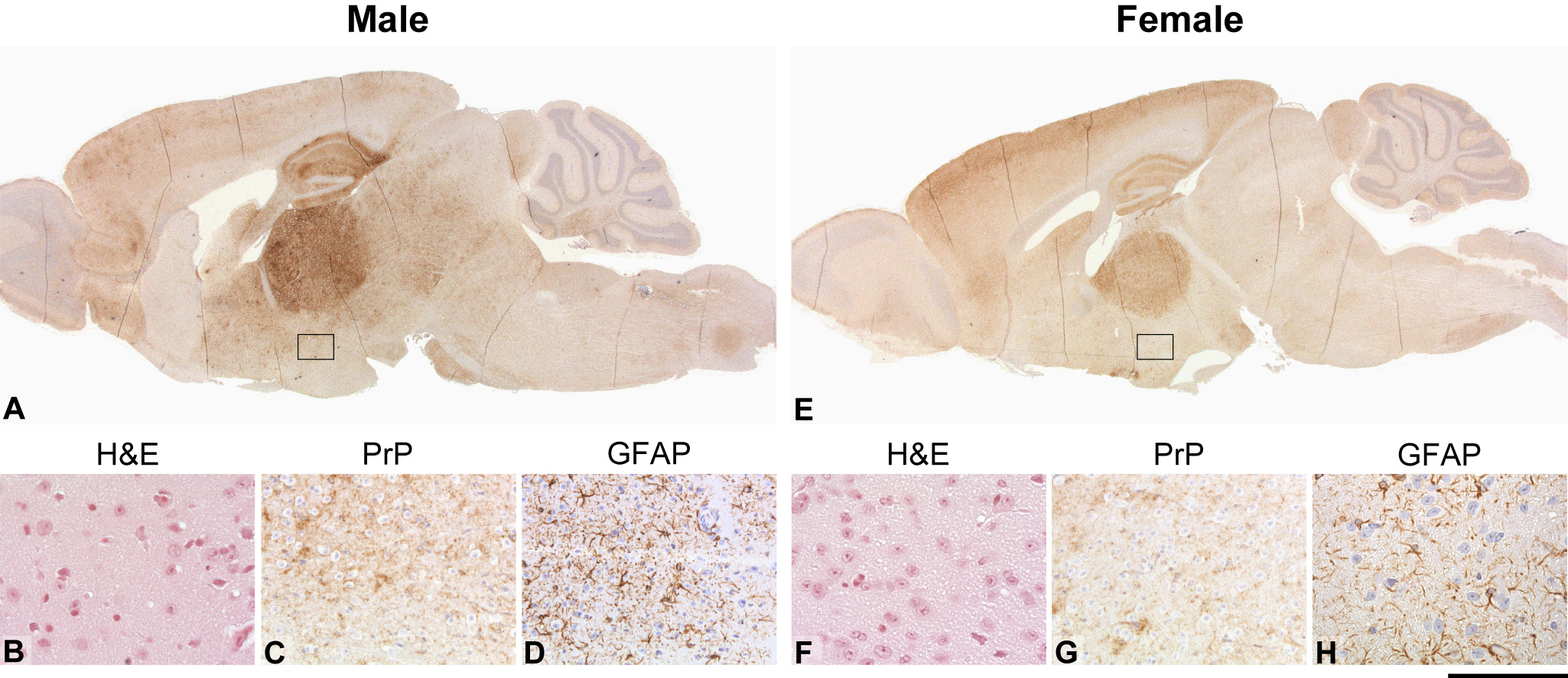

Supplement: Figure S1 — Histological features of Chandler/RML inoculated male and female C57BL/6 mice. Comparison of histological features between male C57BL/6/(left panel, A–D) and female C57BL/6 mice (right panel, E–H) inoculated with Chandler/RML prions. Panels A and E show the PrPSc distribution in a cross section of the brain and panels B–D and F-H are higher power views of the hypothalamus (boxed area). (B and F) mild spongiosis (C and G) synaptic deposition of PrPSc (D and H) gliosis. Overall, the pattern of PrPSc distribution, spongiosis or gliosis, shows no difference between both groups. Scale bar corresponds to 2 mm (A, E), 80 µm (B, F) or 160 µm in all other panels. (JPG) [file pone.0028741.s001.jpg]

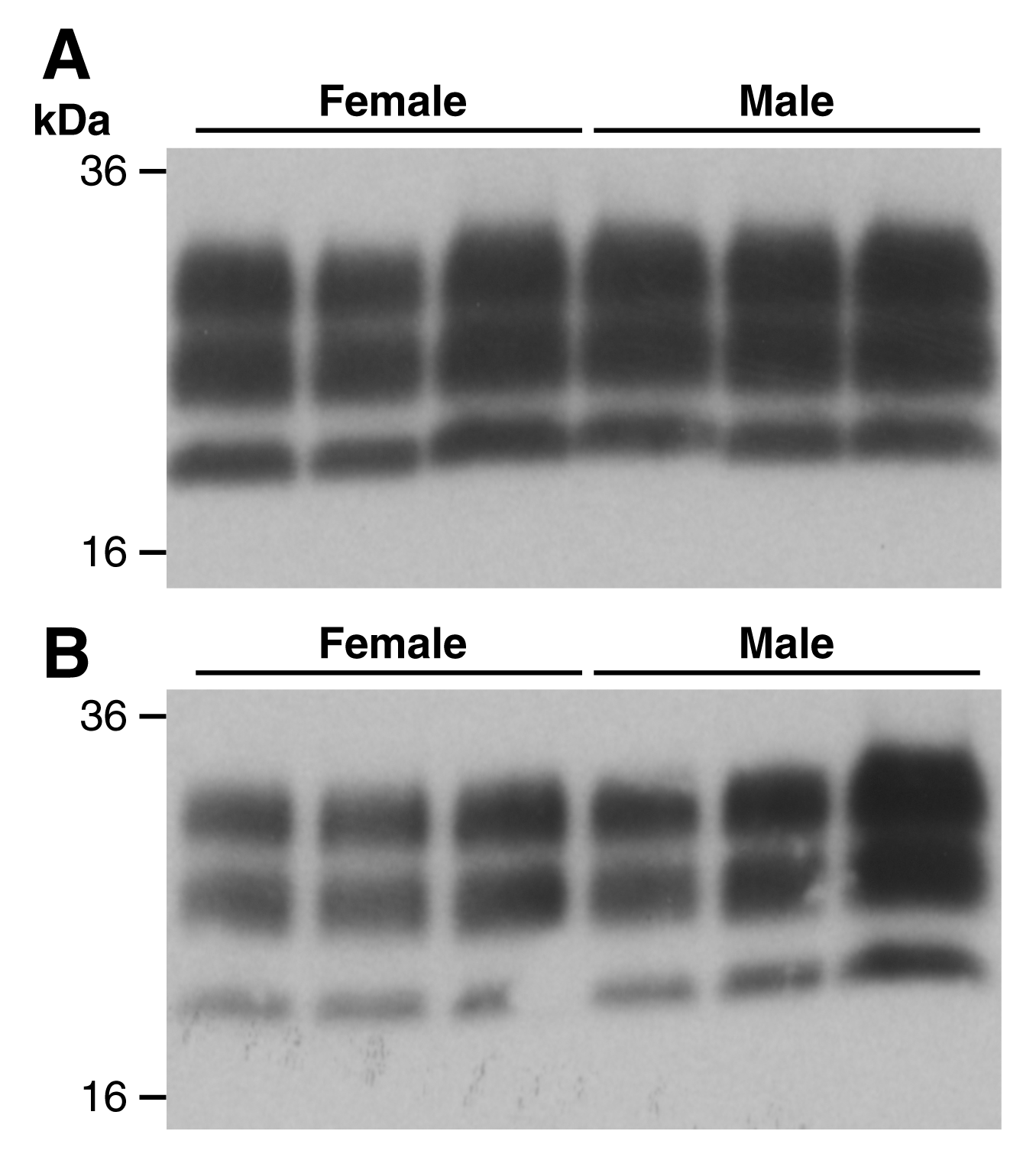

Supplement: Figure S2 — Western blot of PrPSc from the brains of C57BL/6 and FVB/N mice following Me7 transmission. Western blot of proteinase-K treated 10% w/v brain homogenates (n = 3 for both males and females) immunoblotted with anti-PrP monoclonal antibody ICSM-35 (D-Gen Ltd, UK). (A) C57BL/6 mice (B) FVB mice. The PrPSc from both male and female brains is characteristic of the Me7 scrapie prion strain and no sex differences are seen. (TIF) [file pone.0028741.s002.tif]

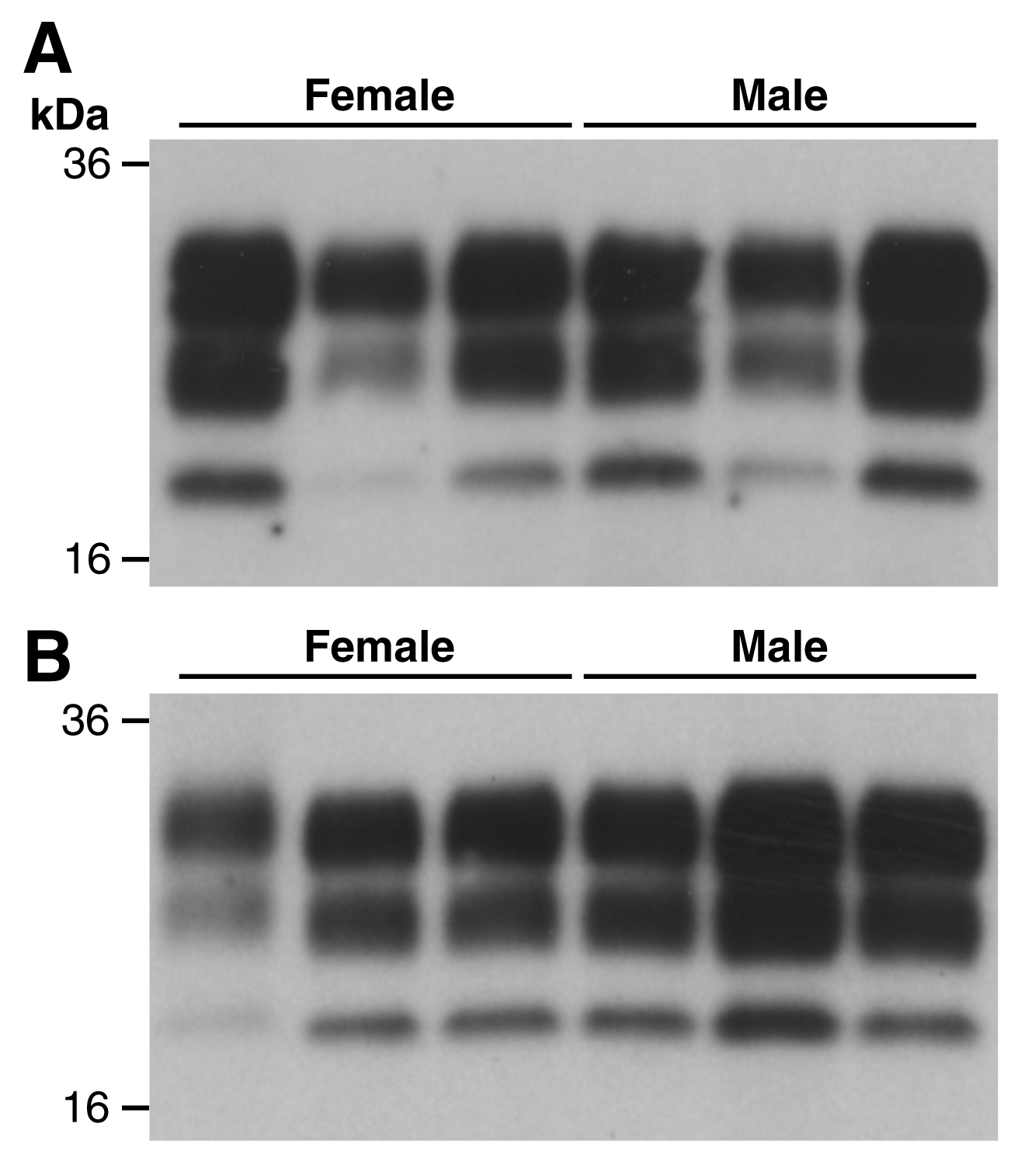

Supplement: Figure S3 — Western blot of PrPSc from the brains of C57BL/6 and FVB/N mice following MRC2 transmission. Western blot of proteinase-K treated 10% w/v brain homogenates (n = 3 for both males and females) immunoblotted with anti-PrP monoclonal antibody ICSM-35 (D-Gen Ltd, UK). (A) C57BL/6 mice (B) FVB mice. The PrPSc from both male and female brains is characteristic of the MRC2 prion strain and no sex differences are seen. (TIF) [file pone.0028741.s003.tif]
